# Modified Diet Use in Adults with Temporomandibular Disorders related to Rheumatoid Arthritis: A Systematic Review

**DOI:** 10.31138/mjr.31.2.183

**Published:** 2020-06-01

**Authors:** Gilheaney Órla, Sibylle Béchet, Margaret Walshe

**Affiliations:** Department of Clinical Speech and Language Studies, Trinity College Dublin, Ireland

**Keywords:** Rheumatoid arthritis, diet, diet modifications, temporomandibular joint, temporomandibular joint disorders

## Abstract

**Objective:**

Individuals presenting with rheumatoid arthritis (RA) frequently experience temporomandibular disorders (TMDs), which can result in limited ranges of mandibular motion, pain and fatigue on jaw function, and impaired mastication. As such, individuals with RA-related TMDs may consume a texture-modified diet in order to reduce the exacerbation of jaw pain and dysfunction, and to increase the ease of oral intake. These softer food options may not contain the recommended nutrients, vitamins, and minerals, and therefore, may not be nutritionally optimal. As unintentional body composition and weight changes are common in individuals with RA, there may be elevated risks of obesity or malnutrition in this patient subgroup. However, minimal researcth has been conducted to investigate the use of modified diets in this cohort, and therefore, the true level of risk to these patients cannot not be adequately determined. The aim of this study was to determine the prevalence of diet modifications in adults presenting with RA affecting the TMJ.

**Methods:**

All available evidence presenting data on adults with RA who consume modified diets was systematically reviewed. A range of electronic databases were searched, including: EMBASE, PubMed, CINAHL, Web of Science, Elsevier Scopus, Science Direct, AMED, The Cochrane Database of Systematic Reviews, and ProQuest Dissertations and Theses A & I. Supplementary Google Scholar, reference list, and grey literature searches were also conducted. Independent reviewers assessed study eligibility, and methodological quality was rated using the Down’s and Black assessment.

**Results:**

One study was eligible for inclusion, and half (50.82%; CI: 37.7–63.86) of individuals with RA in this study consumed a modified diet. This study was rated to be of moderate quality. The primary limitation of this review was the lack of studies on this topic which were available for inclusion.

**Conclusions:**

Although from clinical practice, it is recognised that adults with TMD related to RA do modify their diets to cope with the functional impairment of TMD, this review confirms that minimal research has been conducted regarding the use of texture modified diets by this population. This is despite concerns regarding unintentional weight changes in this patient group. Further research investigating this area is warranted in order to improve patient outcomes and experience of care.

## INTRODUCTION

Rheumatoid arthritis (RA) is an autoimmune disease which negatively impacts on the structure and function of multiple body systems in up to 3% of the global adult population.^1–2^ As such, RA is one of the leading causes of progressive physical disability, and is typically associated with body-wide, functional and physiological implications, reduced health-related quality of life (HRQOL), socioeconomic burdens, and increased patient morbidity and mortality.^1–5^ RA is characterised by progressive remissions and exacerbations of immune-mediated inflammation of multiple symmetrical synovial joints, hyperplastic antibody production, and joint effusion.^6–7^ Although RA typically affects weight-bearing joints,^8^ secondary presentations can include the jaw/temporomandibular joint (TMJ).

RA has a distinct pathological effect on the structure and function of the articular, osseous, and muscular aspects of the entire TMJ complex, ranging from cellular and morphological changes, to widespread myalgia of the supporting muscles of mastication,^9–10^ and even potential destruction of the mandibular condyle.^11^ These changes in TMJ structure and function result in the development of temporomandibular disorders (TMDs) in up to 84% of adult patients presenting with RA,^12–15^ with signs and symptoms including restricted ranges of mandibular motion, pain on mandibular functioning, and impaired mastication across a range of consistencies.^16^ As such, these physiological difficulties may result in adults with RA altering their diet to include greater levels of soft foods which are easier to consume, yet which may be deficient in certain essential vitamins, minerals, nutrients, and calories, and which therefore may lead to unintentional weight changes.

It is well established that the systemic disease processes involved in the pathogenesis of RA themselves may impact negatively on typical nutrition, with the potential for a range of negative secondary consequences, including: weight loss and malnutrition, rheumatoid cachexia, and even obesity.^17–18^ Weight loss leading to malnutrition is highly prevalent in up to 71% of adults with RA, with reduced amounts of fat-free mass found at high levels, even in individuals with low RA disease activity.^19^ It has been suggested that low body mass index (BMI) is a crucial predictor of worse future clinical outcomes, with negative implications on levels of muscle mass and joint destruction.^20^ Therefore, malnutrition and weight loss may be contributing factors to increased morbidity and mortality in adults with RA, with some research hypothesising direct links between increased malnutrition and the presence of RA-related TMDs.^20–21^

In addition to weight loss and malnutrition, metabolic perturbations associated with RA can also result in profoundly reduced muscle mass in the presence of stable or increased body fat.^22–24^ These changes in body composition are known as rheumatoid cachexia and they occur in up to 67% of adult individuals with RA.^25–26^ Despite these dramatic perturbations, it is yet often difficult to detect changes in body composition as the increased or stable body fat levels and RA-related systemic inflammation may mask the muscle wastage, resulting in stable BMIs.^22,27^ However, rheumatoid cachexia has long since been linked to uncontrolled RA disease processes, and is often associated with negative systemic, functional, and psychosocial consequences.^25^

Beyond weight loss and cachexia, increased levels of visceral body fat, central obesity, and waist circumference are also frequently documented in adults presenting with RA,^28,29^ with more than 60% having BMIs above recommended levels,^26^ and up to 57% of these individuals presenting as overweight or even clinically obese.^26,30,31^ Controversy has historically surrounded the investigation of obesity in adults with RA, with conflicting debates regarding the contribution of obesity to the development of RA and the relative effects of levels of body fat on joint function.^18,26,32,33^ Despite this ongoing debate however, there are established links between higher levels of body fat in adults with RA and coronary disease, type 2 diabetes inflammation, insulin resistance, medication resistance, and endothelial dysfunction,^19,26,28,34^ and it is well documented that obesity in adults with RA leads to decreased HRQOL, functional capacity, and increased pain and inflammatory activity,^26,30,34–36^ while individuals with RA who are obese have a 40% lower chance of achieving remission status, and a 50% lower chance of sustaining this status, as compared to those who are not obese.^28^ Also, at a societal level, research has suggested that obesity associated with RA larger medical costs, longer hospital stays, and decreased workforce productivity.^30^ Therefore, in light of these findings, it is evident that the investigation and pro-active prevention of unintentional weight gain in adults with RA is worthy of significant clinical and research attention in order to minimize the risk of negative secondary systemic, psychosocial, and functional sequelae.^26^

As RA may induce a range of unintentional changes in body composition and bodyweight, it is concerning that individuals with RA affecting the TMJ may also be altering their diets to include softer foods, which may not be nutritionally optimal, as this behaviour may represent further risks to nutrition and body status. Despite the potentially elevated risks of undesirable weight changes in adults presenting with RA who consume texture-modified diets, as compared to those without TMJ difficulties, limited research has been conducted on this topic. Minimal evidence exists regarding the prevalence of modified diet use or the methods by which these modifications are achieved, and therefore, cohort-specific nutritional advice to reduce secondary systemic consequences is not currently available. Also, the true contribution of consumption of a modified diet to changes in body composition is not known, due to the myriad of wider concerns relating to malnutrition, cachexia, and/or obesity. The aim of this research was to conduct a systematic review of all available evidence pertaining to the prevalence of consumption of texture modified diets by adults presenting with RA affecting the TMJ.

## MATERIAL AND METHODS

This systematic review was conducted in accordance with the Preferred Reporting Items for Systematic Reviews and Meta-Analyses (PRISMA) statement.^37^

### Eligibility Criteria

All available published and unpublished evidence providing prevalence data regarding the consumption of texture modified diets by adults with RA affecting the TMJ were eligible for inclusion. No secondary restrictions regarding recruitment locations/research settings, publication date, gender, age of onset, disease duration, or disease severity were applied. Case reports were excluded due to critique regarding their typical levels of methodological quality and levels of evidence. Primary studies were also excluded if they presented data relating solely to participants with a history of congenital, traumatic, carcinogenic, or neurological conditions affecting the oral, maxillofacial, or head and neck area.

### Data Sources

Two independent reviewers employed a systematic search strategy across 9 electronic databases, including: EMBASE, PubMed, CINAHL, Web of Science, Elsevier Scopus, Science Direct, AMED, The Cochrane Database of Systematic Reviews, and ProQuest Dissertations and Theses A & I Databases. This sensitive search strategy accounted for database-specific indexing factors such as filters, key-text terms, and Medical Subject Headings, as appropriate. The Zotero (www.zotero.org) bibliographic program was used to collate all retrieved results, and 2 independent reviewers deleted duplicates and screened all identified records to exclude obviously irrelevant articles. The senior author also conducted hand-searches of the annual scientific meetings of the International Association for Dental Research (published in the *Journal of Dental Research*) and the American College of Rheumatology (published in *Arthritis and Rheumatology*), the Google Scholar database, grey literature, and reference lists of studies ultimately included in the meta-analysis. Identified articles which were eligible for inclusion were subsequently analysed by reviewers.

### Data Extraction Process and Data Items

A previously piloted electronic form^16^ was used in data extraction by 2 independent reviewers. Full consensus was reached regarding data extracted, although a third independent author was available to mediate disputes, if required. If data from primary studies published within the previous 10 years was found to be missing/unclear, primary authors were contacted using standardised email templates.^16^ Studies were excluded in cases of no response from authors following 2 contact attempts.

### Assessment of Methodological Quality

Assessments of methodological quality were conducted by 2 independent reviewers, with a third available to mediate disputes, if required. Ratings were completed using the Down’s and Black assessment of methodological quality.^38^

### Summary Measures and Synthesis of Results

Descriptive analysis was conducted using the Microsoft Excel platform in order to describe the characteristics of included studies.

## RESULTS

### Study Identification

**Figure 1.**
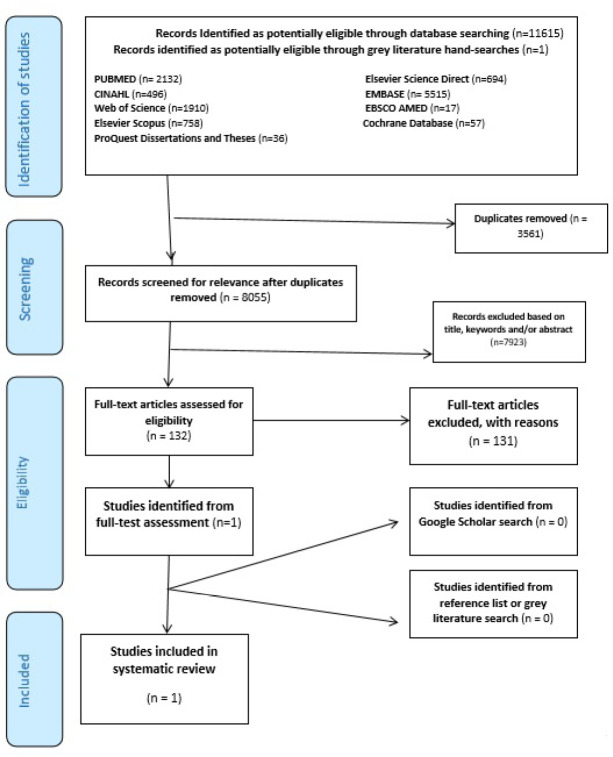
PRISMA Diagram.

Systematic searches of electronic databases initially yielded 11616 records (*Figure 1*). Duplicates were then deleted using the Zotero program, and 2 independent reviewers assessed the eligibility of 8055 articles based on their abstracts, titles, and key words. In total, 132 full-texts were examined in detail, leading to the exclusion of 131 records. No additional articles were identified via supplementary Google Scholar, reference list, or grey literature searches. One article was ultimately included in the analysis.

### Study Characteristics

Study characteristics are displayed in *Table 1*.

**Table 1.** Characteristics of Included Study.

**Citation**	Bessa-Nogueira et al.^39^
**Year of publication**	2008
**Region from which participants were recruited**	Brazil
**Setting from which participants were recruited**	Rheumatology Department, University Hospital
**Year of recruitment**	December 2003 – December 2004
**Study design**	Descriptive observational
**No. of RA patients**	61
**Female : male ratio**	9:01
**Mean age (range) of RA patients (years)**	NA (24–76)
**Mean age (range) of onset (years)**	NA (NA-NA)
**Mean disease duration (range) (years)**	NA(NA-NA)
**Main outcome**	Diet modifications: 50.82%
**Sources of assessment data**	Clinical exam, patient interviews, Health Assessment Questionnaire, visual analog scales

The design of the included study was descriptive observational, with research conducted in the Rheumatology Department of a Brazilian university hospital.^39^ Data was gathered using a range of subjective outcome measurement tools, including: clinical examinations, patient interviews, visual analog scales, and the Health Assessment Questionnaire.^40^

### Description of Participant Demographics

Data regarding 61 individuals presenting with RA was extracted from the 1 study included in this systematic review, with a mean age of 44.7 years of age (range: 24–76 years of age) (*Table 1*). Analysed data was characterised primarily by females (n=55) as compared to males (n=6), with a female: male ratio of 9:1.

### Assessment of Methodological Quality of Included Studies

Independent reviewers reached 100% consensus regarding assessments of methodological quality. The included study was awarded a score of 11 points out of a possible 16, indicating a rating of moderate quality. Contributing to positive ratings were the clear description of aims, objectives, outcomes, and participant demographics, and the use of valid and reliable outcome measurement tools. Negative ratings centred primarily on the lack of discussion or analysis of principal confounding factors, and the lack of justification of sample size.

### Prevalence of Diet Modifications

Based on data extracted from this study (n=61),^39^ the prevalence of diet modifications was estimated to be 50.82% (confidence interval: 37.7–63.86).

## DISCUSSION

This systematic review established that minimal research has been conducted into the consumption of modified diets by adults presenting with RA-related TMDs. This mirrors the typically limited clinical attention to this issue, with this combined lack of academic and practical focus potentially impacting negatively on therapeutic outcomes and patient recovery. This finding is significant as, at baseline, the risk of incurring unintentional body composition changes is elevated among adults with RA, in comparison to the general public. As such, if individuals with RA also consume a nutritionally sub-optimal modified diet due to TMJ degeneration, they may face a myriad of additional adverse consequences on health and functioning.

For example, individuals with RA-related TMDs may consume modified diets of a soft, minced-moist, or smooth pureed consistency in order to ameliorate masticatory impairments. However, without adequate nutritional guidance, these diets may be characterised by convenience foods high in fat, salt, and sugar, which if consumed frequently, can increase the individual’s fat composition, body weight, and BMI. Within the general population, higher levels of body fat increase the risk of developing a range of endocrine, metabolic, and vascular conditions, among others. However, in adults with RA, having a higher BMI incurs even graver consequences. For example, higher levels of body fat place greater stress on weight-bearing joints, with these increases in joint loading augmenting pain and dysfunction. Additionally, at a molecular level, fat tissue releases excess cytokines which circulate throughout the vascular system, initiating, perpetuating, and amplifying low-grade inflammation and perceived RA severity.^41^

At the opposite end of the spectrum, it is also possible that such individuals may reduce their oral intake or modify their diets to consume calorie deficient meals, resulting in weight loss and further systemic difficulties. For example: significant weight loss may result in sarcopenia, muscle atrophy, reduced wound healing, and osteoporosis, with increased joint damage and dysfunction.^42,43^ Also, the consumption of modified diets which exclude hard, tough, or crunchy, fibrous foods (eg, whole-wheat bread, baked potatoes, nuts, or raw vegetables) may perpetuate or exacerbate the gastrointestinal motility issues with which patients with RA frequently present, thus increasing systemic dysfunction and discomfort.^44^ As such, in light of these potential adverse outcomes, further research into the provision of effective management and guidance regarding the appropriate consumption of nutritionally optimal modified diets is essential, with the ultimate goal of reducing the substantial psychosocial, medical, and economic costs associated with both RA and unhealthy bodyweight levels.^45^

### Limitations

The main limitation of this systematic review was that just one study met the pre-specified eligibility criteria, leading to authors being unable to conduct a meta-analysis of prevalence figures. True prevalence rates regarding the consumption of texture modified diets by adults presenting with RA-related TMDs may be different to that presented in this study and further research investigating modified diet use should be carried out in order to advance the professional knowledge base.

### Recommendations

This study concludes with the recommendation that future research investigates the following areas:

- The true prevalence of modified diet use by this patient group in order to establish epidemiological data to assist in care planning;
- The typical oral intake of adult individuals presenting with RA-related TMDs in order to assess if the nutritional and calorific content of these diets is sufficient and in accordance with national and international guidelines for healthy adults of a similar age, BMI, and activity level;
- The spectrum of possible systemic, nutritional, and psychological risks incurred by consuming a texture modified diet in the context of rheumatic disease; and
- The need for, and methods of delivery of, specialist multi-disciplinary care to ensure that adequate oral intake is sustained if elevated risk to nutritional status is found to be present.

## CONCLUSIONS

This study has highlighted the lack of research which has been conducted on the use of diet modifications by adults presenting with RA-related TMDs, despite the identified risks to nutritional status and body composition within this vulnerable population. Therefore, subsequent investigations are warranted, with particular emphasis on the frequency and methods of use and nutritional values of these modified diets, to allow for exploration of potential additional risks which this behaviour may represent to patients already compromised nutritional status.
